# Large-scale manual curation and harmonization of metadata from metagenomic and cancer genomic repositories: challenges and solutions

**DOI:** 10.1093/database/baag027

**Published:** 2026-05-22

**Authors:** Kaelyn Long, Kai Gravel-Pucillo, Levi Waldron, Sean Davis, Sehyun Oh

**Affiliations:** Institute for Implementation Science in Population Health, City University of New York School of Public Health, New York, NY, United States; Department of Epidemiology and Biostatistics, City University of New York School of Public Health, New York, NY, United States; Institute for Implementation Science in Population Health, City University of New York School of Public Health, New York, NY, United States; Department of Epidemiology and Biostatistics, City University of New York School of Public Health, New York, NY, United States; Institute for Implementation Science in Population Health, City University of New York School of Public Health, New York, NY, United States; Department of Epidemiology and Biostatistics, City University of New York School of Public Health, New York, NY, United States; Departments of Biomedical Informatics and Medicine, University of Colorado Anschutz School of Medicine, Denver, CO, United States; Institute for Implementation Science in Population Health, City University of New York School of Public Health, New York, NY, United States; Department of Epidemiology and Biostatistics, City University of New York School of Public Health, New York, NY, United States

## Abstract

Public omics repositories contain vast amounts of valuable data, but their metadata suffers from extreme heterogeneity, unstandardized terminologies, and quality issues that severely limit data reusability and cross-study integration. While prospective metadata standards exist, the majority of published omics data remain in non-standardized formats requiring retrospective harmonization. We performed comprehensive manual curation and harmonization of metadata, such as participant characteristics and study conditions, from 212 027 omics samples across 468 studies in two repositories: *curatedMetagenomicData* (93 studies, 22 588 samples) and cBioPortal (375 studies, 189 438 samples). Through systematic ontology mapping, we consolidated redundant, dispersed information into far fewer harmonized columns, reduced unique values, and increased the completeness of major attributes. This curation process revealed common metadata quality issues, including typos, inconsistent terminologies, misplaced values, conflicting annotations, and inappropriately merged information across attributes. We document the challenges, decisions, and solutions during this large-scale metadata harmonization. The harmonized metadata, accessible through the *OmicsMLRepoR* Bioconductor package, enables repository-wide queries and cross-study analyses previously challenging with heterogeneous metadata. Our experience provides practical guidance for similar curation efforts and demonstrates the value of investing in retrospective metadata improvement for existing public omics resources.

## Introduction

The exponentially growing volume of public omics data presents opportunities for cross-study analysis, thereby improving statistical power and enhancing generalizability [1,2]. Several examples have demonstrated significant advantages in understanding diseases through cross-study analysis, including the identification of disease signatures [3], enriched pathways [4], and potential treatments [5,6]. This potential requires standardized metadata that enable data identification, integration, and interpretation; however, their inherent complexity and heterogeneity pose significant challenges for standardization and validation [7–9]. Despite numerous calls for improved metadata standards and several proposed frameworks [9–11], most published omics data still contain heterogeneous, unstandardized metadata that severely limits their usability.

Several challenges exist in developing metadata that complies with the FAIR (Findable, Accessible, Interoperable, Reusable) principles [10–12]. First, there are too many data standards [13]. Some existing data standards impose overly granular specification requirements that exceed practical implementation capacities [14]. Metadata augmentation efforts often focus on developing new data standards [15, 16] rather than implementing existing ones. Second, biological data collection and management commonly involve unstandardized values, such as curator-defined abbreviations and drug brand names. The same or similar concepts are frequently categorized or labelled differently across studies. Ontologies provide a solution by offering standardized vocabularies and identifiers for domain-specific concepts, facilitating data organization, integration, exchange, and reuse in machine-readable formats. However, ontologies are not widely used in biomedical data collection due to several practical barriers, including their complexity and training requirements, time constraints during data collection, and a lack of convenient tools. Third, most biomedical repositories lack quality and sanity checks at the value level. Even common human errors, such as typos or values under the wrong attribute that can be systematically recognized and corrected, are common in public data.

The research community has explored multiple approaches to address these issues, each with distinct strengths and limitations for different use cases (Table 1). First, *prospective standardization frameworks* have made significant advances in specific domains (Table 2). The Observational Medical Outcomes Partnership (OMOP) Common Data Model [17, 18] provides comprehensive standardization for observational health data, particularly electronic health records, enabling large-scale epidemiological studies across healthcare systems. Health Level Seven Fast Healthcare Interoperability Resources (HL7 FHIR) [19] offers flexible, web-based standards for health information exchange in clinical settings. Domain-specific frameworks like ISA (Investigation-Study-Assay) [20] and reporting guidelines such as MIAME (Minimum Information About a Microarray Experiment) [21], MINSEQE (Minimum Information about a high-throughput Nucleotide SeQuencing Experiment), and MIxS (Minimum Information about any Sequence) [22] provide valuable guidance for metadata collection in experimental contexts. These frameworks were designed primarily for prospective use during data collection, and their adoption by the research community is central to advancing FAIR data practices. Recent work also demonstrates that community standards can be leveraged retrospectively—e.g. encoding reporting guidelines as machine-actionable templates [23] and using them to guide generative AI systems that rectify legacy metadata in repositories such as BioSample and GEO [24,25], which are very promising. The challenge that remains, and which the present work addresses, is that a substantial portion of already-published omics data was generated before such standards were widely available and still requires harmonization to become broadly reusable. Second, *automated rule-based harmonization* approaches have been developed to programmatically standardize metadata using predefined transformation rules [26–28]. These methods excel when metadata follows predictable patterns and when data are structured consistently. However, they struggle with the extensive edge cases and context-dependent interpretations common in real-world omics metadata. For instance, distinguishing whether ‘stage II’ in a free-text field refers to disease staging, treatment line, or experimental phase requires contextual understanding that simple rules cannot capture. Recently, large language model (LLM)-based approaches are particularly active and promising in automated harmonization [29–31]. Verbitsky *et al*. [31] and others have demonstrated that LLMs can extract and standardize metadata from unstructured text with encouraging results, especially when grounded in community standards or ontologies. The remaining technical challenges that motivate continued methodological work include performance degradation on out-of-vocabulary terms, hallucination risks where models generate plausible but incorrect standardized terms [32–34], and limited capacity to resolve cross-attribute logical inconsistencies that depend on domain knowledge (e.g. recognizing that ‘prostate cancer’ paired with ‘female’ sex represents an error) [35, 36]. Hybrid approaches that combine automated methods with curator oversight or that incorporate domain knowledge into LLM prompts are advancing rapidly and are likely to play an increasingly central role in repository-scale harmonization. However, these hybrid methods remain under development, still requiring substantial human oversight [37], and have been applied primarily to small-scale pilot studies rather than repository-level harmonization [25,38]. *Manual curation efforts* by other investigators have successfully harmonized metadata for specific use cases. Adhikari *et al*. [39] demonstrated effective data pooling from cohort studies through manual harmonization but focused on 3–10 carefully selected studies with relatively homogeneous data structures. Biedermann *et al*. [40] standardized pulmonary hypertension registry data to the OMOP CDM but required substantial domain expertise and were limited to a single disease area.

**Table 1:** Comparison of metadata harmonization approaches across repositories.^a^

Approach	Scale	Automation	Accuracy	Use case	Limitations
Rule-based	Medium	High	Medium	Structured data	Fails on edge cases
LLM-based	Medium	High	Variable	Text extraction	Hallucination risk
Hybrid	Small-medium	Medium	High	Mixed data	Under development
Manual curation (prior)	Small	Low	High	Well-defined datasets	Labor intensive
Manual curation (this work)	Large	Low	High	Heterogeneous legacy data	Resource intensive

a Different approaches to metadata harmonization are evaluated across five key dimensions. ‘Scale’ refers to the volume of data that can be practically processed (small: <10 000 samples; medium: 10 000–100 000 samples; large: >100 000 samples). ‘Automation’ indicates the degree of manual intervention required (low: predominantly manual review; medium: semi-automated with human oversight; high: minimal human intervention). ‘Accuracy’ reflects the reliability of harmonization outputs (medium: 70–85% correct mappings; high: >85% correct mappings; variable: accuracy depends on data characteristics). ‘Use case’ describes the optimal application scenario for each approach. ‘Limitations‘ identify the primary challenges or constraints of each method. Prior manual curation efforts typically focused on smaller, well-curated datasets, whereas this work applies manual curation at scale to handle diverse, heterogeneous legacy data across multiple repositories.

**Table 2:** Comparison of OmicsMLRepo with existing biomedical data standardization frameworks.^a^

Framework	Application timing	Primary domain	Target scale	Infrastructure requirements	Primary use case	Key limitation for legacy data
OMOP CDM	Prospective	Clinical EHR	Institution-wide	High (ETL pipelines, CDM implementation)	Clinical data standardization	Requires data re-engineering
HL7 FHIR	Prospective	Clinical exchange	Health system	Medium-high (API infrastructure)	Interoperable health data exchange	Standards must be adopted at source
ISA	Prospective	Laboratory and experimental	Study/experiment	Medium (ISA tools, metadata templates)	Structured metadata at the collection	Not designed for post hoc harmonization
OmicsMLRepo	Retrospective	Multi-omics research	Cross-repository	Low (no source modification required)	Legacy data harmonization across repositories	Resource-intensive manual curation

a This table positions OmicsMLRepo within the landscape of clinical and experimental data standardization approaches, highlighting fundamental differences in design philosophy and application. ‘Application timing’ distinguishes whether frameworks standardize data prospectively (at the point of collection) or retrospectively (harmonizing existing legacy datasets). ‘Primary domain’ specifies the data type or research area. ‘Target scale’ indicates the typical scope of deployment. ‘Infrastructure requirements’ describes the computational and organizational resources needed for implementation, with ‘High’ requiring enterprise-level database systems and ETL pipelines, ‘Medium’ requiring dedicated tools and templates, and ‘Low’ requiring minimal technical infrastructure at data sources. ‘Primary use case’ identifies the main application scenario. ‘Key limitations for legacy data’ highlights challenges when applying each framework to existing datasets. Unlike prospective frameworks that require standardization during data generation and substantial infrastructure investment, OmicsMLRepo enables retrospective harmonization of heterogeneous multi-omics metadata across existing public repositories without modifying source databases, making it uniquely suited to integrating legacy research data at scale. ETL = Extract, Transform, Load; CDM = Common Data Model; ISA = Investigation-Study-Assay.

These collective efforts highlight major conflicts in the field: automated methods offer scalability but sacrifice accuracy and contextual judgement that complex, heterogeneous biomedical metadata demands. Manual curation provides this depth but has historically been limited to narrow domains or small sample sizes. To the best of our knowledge, no existing approach provides a systematic methodology for retrospectively harmonizing metadata across hundreds of independently conducted studies spanning multiple omics types, each with distinct data models, terminologies, and curation practices. This gap is particularly problematic given that the vast majority of valuable public omics data already exist in non-standardized formats, and achieving consensus for prospective standards across the diverse omics research community remains challenging.

To address this gap, we introduce the OmicsMLRepo project—a metadata harmonization initiative focusing on the practical and retrospective harmonization of already-collected research data across research-derived omics repositories. In this project, we define metadata harmonization as a systematic process of 1) identifying conceptually similar information scattered across different attributes and studies; 2) consolidating this information into standardized attributes with consistent naming and structure; 3) mapping values to established ontologies and controlled vocabularies; 4) correcting data quality issues including typos, inconsistent terminologies, and logical errors; and 5) resolving conflicts in annotations. We aim to transform heterogeneous, study-specific metadata into a unified representation that enables cross-study queries and integration while preserving granular information through flexible attribute structures. We report our experience performing large-scale, retrospective manual curation and harmonization of sample metadata from two major research-derived omics repositories: *curatedMetagenomicData* (cMD) [41] and *cBioPortalData* [42] Bioconductor packages. The cMD package contains 22 588 whole metagenomic shotgun sequencing datasets from 93 studies, all uniformly processed and accompanied by manually curated metadata. While cMD metadata were curated by experienced curators, they have not been harmonized across studies and remain plagued by low-quality issues, such as typos and redundancy. The *cBioPortalData* package provides access to data from the cBio Cancer Genomics Portal (cBioPortal) [43–45]. cBioPortal is a free, open-source platform for exploring and analysing cancer genomics data, providing large-scale cancer genomics datasets that include molecular and subject/sample-level metadata from various studies and sources. However, cBioPortal’s sample metadata is extremely heterogeneous and lacks standardization. Different studies use distinct, often arbitrary terms and abbreviations for the same entities, making it challenging to identify samples across studies using metadata.

Our work covers the full curation lifecycle from schema reconciliation through ontology incorporation to value standardization, and demonstrated the improved accessibility of the large-scale harmonized metadata through examples of repository-wide summary statistics. We developed the *OmicsMLRepoR* Bioconductor package to provide easy access to and manipulation of harmonized metadata. The improved metadata quality through OmicsMLRepo project will enable researchers to effectively find and utilize relevant datasets, reduce redundant data generation, facilitate cross-study analyses, improve AI/ML-readiness of public omics data, advance research reproducibility, and accelerate discoveries.

## Methods

**Datasets**. We harmonized metadata from research-derived omics (non-EHR) data available through *curatedMetagenomicData* (cMD, version 3.8.0) and *cBioPortalData* Bioconductor packages that did not adhere to prospective metadata standards during data collection. For *cBioPortalData*, we captured sample metadata (e.g. participant characteristics and study conditions) on 8 May 2023, and selected core attributes for harmonization based on their completeness, prevalence across data resources, and clinical relevance. All the cMD attributes were harmonized. To minimize data duplication and maintain integration with existing user workflows, our harmonized metadata tables are aimed to be redistributed through the source packages (*curatedMetagenomicData* and *cBioPortalData*). Users can access harmonized metadata as additional data objects within these packages using the OmicsMLRepoR::getMetadata() function, while the original metadata remains available for reference.

**Exploratory data analysis and schema development**. Before establishing our harmonization schema, we conducted comprehensive exploratory data analysis (EDA) to understand the heterogeneous structure of metadata across studies. Our EDA process involved examining both attribute names and their corresponding values, as datasets exhibited significant structural differences in how they organized conceptually similar information. For example, one study might consolidate all treatment information into a single column containing medication name, type, dosage, and administration frequency as free text, while another study might decompose the same information into separate binary attributes for each specific treatment. We systematically identified and grouped related information scattered across different attributes, which enabled us to interpret individual values within their proper context, apply consensus-based harmonization strategies, and minimize redundancy in the final harmonized dataset. This empirically grounded approach, necessitated by the lack of standardization in legacy data, enabled us to develop a data model that accurately represents real-world data characteristics and facilitates the integration of diverse data sources without discarding study-specific information.

**Extract, Transform, and Load (ETL)**. During the data extraction step, we manually reviewed and identified information dispersed and redundant across multiple attributes, collecting all unique values from them. These values were manually curated to ontology terms, and the resulting per-attribute ‘*original value: ontology term*’ map was used for value transformation. For numeric attributes, we separated the numeric and unit components.

The harmonization process involved systematic data cleaning and ontology mapping procedures:

- Data cleaning
  - Multiple values assigned to a single cell were delimited using semicolons unless otherwise specified.
  - Binary columns (e.g. presence/absence) were converted to descriptive values.
  - Empty strings and non-interpretable values were assigned *NA*.
  - When multiple values at different resolutions were available for a given term, we consolidated to the most specific information.
- Ontology mapping
  - Full names were identified for all abbreviations.
  - Unique values were mapped to appropriate ontology terms [46].
  - Original values were updated using manually created ‘*original value: ontology term*’ pairs.

We also revised inaccurate and conflicting information through cross-comparison and literature reviews. When there were multiple ontologies for a single term, we prioritized them based on two criteria: 1) one leading to fewer ontologies per attribute and 2) one that provides better interoperability with other resources.

For each metadata attribute, all unique original values were manually mapped to curated terms. These curation maps include three fields: original_value, curated_ontology_term, and curated_ontology_term_id. Exceptions included partially different column names (e.g. curated_antibiotics), additional columns when needed (e.g. curated_age_min and curated_age_max for age group curation), and newly created columns (e.g. curated_target_condition). The consolidation process and resulting data model are summarized as merging schema tables (Supplementary Tables 1–2) and data dictionaries (Supplementary Tables 3–5), respectively.

**Conflict resolution**. Harmonization of metadata from multiple sources inevitably revealed conflicting annotations for the same samples; e.g. we identified cases where the same patient sample appeared in multiple cBioPortal studies with different sex annotations, or where multiple samples from the same participant had inconsistent ancestry labels. We developed a systematic approach to resolve these conflicts based on the nature and cause of discordance. First, we distinguished between systematic and random errors, as this determines the appropriate correction strategy. Systematic errors (e.g. data entry error accidentally swiping Yes/No) were corrected at the source level when possible. Second, we applied logical consistency checking through cross-attribute validation based on established medical knowledge. For instance, combinations such as ‘prostate cancer’ with ‘female’ sex represent logical impossibilities that flagged errors requiring correction. Third, for attributes that remain constant over time (e.g. sex assigned at birth and genetic ancestry), we applied consensus-based harmonization using a majority rule. When multiple samples from the same patient contained conflicting values for such attributes, we selected the value appearing most frequently. If no majority existed, we replaced values with *NA*. This approach enabled us to infer sex for 422 additional samples and correct 39 incorrect values in the cBioPortal metadata. We carefully limited this consensus-based strategy to attributes that cannot change over time, excluding dynamic/evolving attributes such as disease stage or treatment status.

**Ontology integration and dynamic value validation**. We leveraged ontologies not only for standardizing values but also for systematic value validation. For each harmonized attribute, we defined one or more ‘dynamic enumeration nodes’ (*dynamic enums*). *Dynamic enums* are selected among the shared ancestors from relevant ontologies (i.e. already captured values). All the descendant terms from dynamic enums automatically constitute the allowed values for that attribute. For example, we designated ‘ancestry category’ (HANCESTRO:0004) as a *dynamic enums* for the ancestry attribute, making all eight child terms and their combinations valid values. When choosing dynamic enums, we carefully evaluated the trade-off between creating categories that are too broad to be useful versus too narrow to accommodate diverse data sources.

We can systematically identify data entry errors by validating values against a pool of allowed values (i.e. descendants of dynamic enums); if values do not belong to the pool, they are likely wrong or miscategorized values. We also validate manual curation results by looking up ontology terms using curated ontology IDs (via the *rols* R package [47]) and comparing them with the curated ontology terms. Updates were frequently made during the sanity check, including correcting typos, incorrect interpretations of abbreviations, incorrect ontology IDs, and mismatches between ontology terms and IDs. This ‘round-trip validation’, as well as the dynamic enum-based validation, enabled us to identify and correct inconsistencies that would have otherwise compromised data quality.

**Flexible attribute structures for heterogeneous data**. The EDA revealed that rigid single-valued schemas could not accommodate the structural diversity across studies. We therefore implemented three attribute types based on their value structure: single-valued attributes (one value per attribute), multi-valued attributes (multiple values per attribute), and composite attributes (multiple related features under a single generic attribute). Multi-valued attributes allow multiple standardized values to be assigned to a single attribute for a single sample (e.g. a patient receiving multiple concurrent treatments). Values are separated by semicolons as delimiters. This design preserves the complete information without requiring separate rows for each value, which would complicate sample-level analyses. Composite attributes enable logical grouping of related features under a single generic attribute. This structure improves conceptual clarity and reduces visual complexity in data tables, enables users to quickly understand the extent of available data, provides flexible representation that can accommodate different numbers of measurements per sample, and allows easy addition of new measurements without restructuring the entire database.

To address granularity mismatches across data sources, we created paired attributes: standard/main attributes containing harmonized terms at an appropriate level for cross-study comparisons, and corresponding ‘*_details’ attributes preserving more specific information. For instance, the ancestry attribute contains eight standardized child terms from HANCESTRO:0004, while ancestry_details preserves additional granular information mapped to extended ontology terms. This dual-level approach eliminates the traditional trade-off between preserving granular detail and achieving broad compatibility, ensuring that no meaningful information is discarded during harmonization.

**Figure 1:**
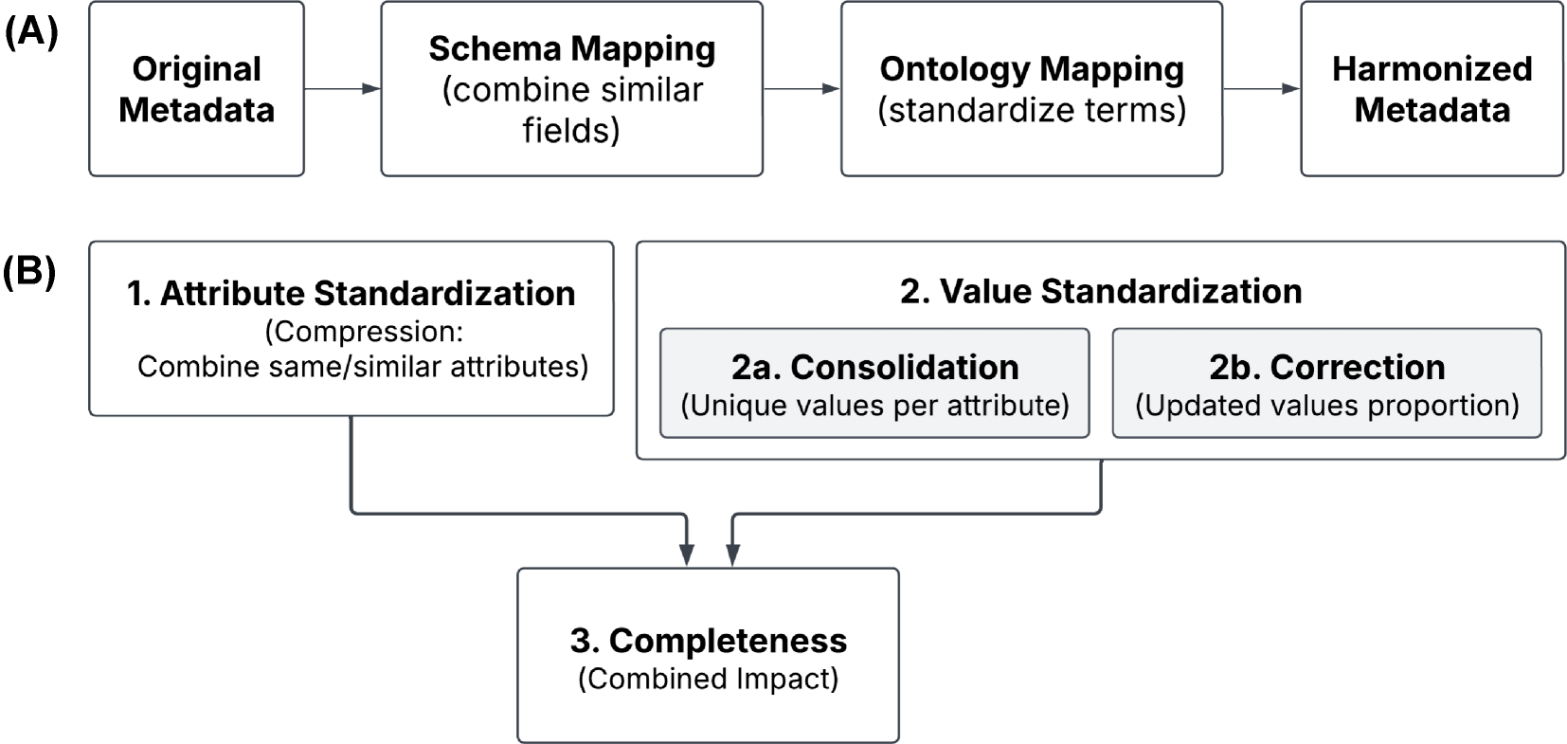
Overview of the harmonization process and quality metrics. (A) Simplified workflow showing the major steps in metadata harmonization. The harmonization process begins with ‘Original Metadata’ from heterogeneous research-driven omics data sources. During the ‘Schema Mapping’ step, conceptually similar attributes are identified and grouped across studies through comprehensive exploratory data analysis, enabling the consolidation of information dispersed across multiple fields with varying structures. The ‘Ontology Mapping’ step standardizes unique values by mapping them to appropriate ontology terms from established vocabularies (i.e. ontologies), while simultaneously performing data validation and quality control. (B) Four metrics used to assess improved metadata quality. The harmonization quality was evaluated using complementary metrics that capture different aspects of metadata improvement: *1. Attribute standardization (compression)* measures how many original attributes were merged into each harmonized attribute; *2-1. Value standardization (consolidation)* quantifies the reduction in unique values per attribute after harmonization; *2-2. Value correction (correction rate)* represents the proportion of values that were updated during curation; *3. Completeness (combined impact)* represents the proportion of non-NA values per attribute.

**Quality assessment framework**. Our manual harmonization processes include quality assessments, such as ‘how we know metadata are inaccurate’ or ‘which ontology terms are appropriate’. The following list addresses practical implementation challenges that we encountered during retrospective harmonization and strategies adopted to handle them:

1. *Identifying inaccurate metadata*. When a value conflicts with our current biomedical knowledge, we consider it inaccurate. This includes cross-attribute logical consistency checks (e.g. ‘ovarian cancer’ and ‘male’ are impossible combinations for a single sample, because males do not have ovaries and cannot develop ovarian cancer) and consensus-based validation across attributes (e.g. multiple samples from a single patient cannot have different biological sex). We also conducted original literature verification and statistical outlier detection (e.g. age >130 years) to identify incorrect metadata.
2. *Selecting appropriate ontology terms*. We used several qualitative rules to guide curators to decide appropriate ontology terms. First, the key consideration is interoperability: minimize the number of ontologies used for a given attribute and choose the ontology used by large existing repositories. For example, we choose NCIt for cancer types to enable integration with the NCI GDC (Genomic Data Commons) portal and Cancer Research Data Commons. Second, we established a prioritization hierarchy for ontology selection, from domain-specific to general-purpose. Examples include UBERON for body site and Gazetteer for country.
3. *Resolving granularity mismatches*. We keep the most specific information while offering widely used, higher-level information separately as needed. Data from different sources often contain information at varying levels of detail; some may use broad categories while others employ highly specific terms. Ontologies help address this granularity mismatch by providing hierarchical structures that allow curators to identify an appropriate level of specificity for harmonization. We accomplished this dual-level approach by creating both standard/main attributes and their corresponding ‘details’ versions. This eliminates the traditional trade-off between preserving granular detail and achieving broad compatibility, ensuring that no meaningful information is discarded during the harmonization process. However, implementing this strategy requires domain knowledge, as terms can often appear in multiple ontological branches, potentially leading to multiple valid ancestor terms. These must be carefully evaluated to select the most appropriate hierarchical path for preserving the original meaning and facilitating harmonization purposes.
4. *Investigate potential reasons for discordance*. It is crucial to distinguish between systematic and random errors, as the cause of discordance determines the appropriate correction strategy. Systematic errors should be addressed by contacting the data providers to correct the source data and prevent future issues. Also, systematic errors should be fixed before applying the majority-rule-based correction. For example, during our harmonization of sex attribute in *cBioPortalData*, we found that 303 of 337 discrepancies were from a single study (*luad_msk_npjpo_2021*) and confirmed that it was a mistake in the original data recording step.
5. *Maintain a consistent naming and formatting schema*. This is essential for scalability, interpretability, and automated processing. First, we suggest using consistent prefixes or suffixes in file and attribute names. Additionally, we recommend making both file and attribute names human- and machine-readable; i.e. avoid using programming language keywords, special characters, and spaces. Consistent value formatting examples include using a single standard NA (not a mix of NULL, missing, -, or 999) and consistently formatting boolean values (e.g. yes/no or true/false).
6. *Quality metrics and validation*. We used the four criteria to assess the improved quality of metadata from semi-structured data (Fig. 1B). However, the interpretation of these quality improvements is context-dependent and may vary depending on the characteristics of the input data. For example, increasing standardization might initially reduce completeness when strict validation rules exclude included-but-non-conformant values. Similarly, improving consistency may reduce the number of unique values as detailed free-text entries are mapped to standardized categorical terms. Different data domains may require distinct evaluation metrics, such as reference range alignment (ensuring clinically meaningful normal/abnormal flags) or the scope of allowed values. While there is no universal, predefined rule for selecting metrics, having appropriate quality metrics is essential for evaluating the harmonization strategy, detecting errors, and iteratively refining the harmonization process to achieve an optimal balance across all quality dimensions while maintaining usability.
7. *Data provenance tracking and documentation*. This benefits both the curators and downstream users. Comprehensive documentation captures not just what was done, but why specific decisions were made during the harmonization process. This contextual information becomes invaluable when original team members leave, when revisiting old projects, or when training new researchers on established protocols. Detailed tracking also enables tracing back through the process when discrepancies or unexpected results emerge. This audit trail enables the identification of exactly where issues occurred and facilitates targeted corrections without requiring the restart of the entire harmonization process.
8. *Normalization strategies*. Curators were trained with demo cases—age and disease harmonization—on ontology basics, resolution of complex cases, and conflict identification. During the post-training period, weekly curation meetings (1 hour) were held to discuss ambiguous cases, new patterns, and ontology updates. The major harmonization work required >1500 person-hours.

**Naming schema and data provenance**. To maintain transparency and traceability, harmonized metadata tables include: 1) columns with ‘original_’ or ‘curated_’ prefixes (in the data provenance version), 2) a ‘last_modified’ timestamp, and 3) a ‘curation_id’, which is a concatenation of other ID columns to create a unique identifier per sample (study_name:participant_id for cMD; study_name:participant_id:sample_id for *cBioPortalData*). The naming schema for data provenance follows this structure:

- curated_{attribute_name} (e.g. curated_age)
- curated_{attribute_name}_{options} (e.g. curated_age_unit)
- curated_{attribute_name}_ontology_term_id
- (e.g. curated_age_ontology_term_id)
- curated_{attribute_name}_source (e.g. curated_age_source): the original attribute name from which the value was captured
- original_{attribute_name}_value (e.g. original_age_value)

**Data dictionary**. The data dictionary describes the major characteristics of the curated fields (Supplementary Tables 3–5). We maintain two formats for the same version: one for curators during the data curation stage and another for the release version presented to end users. The main difference is compactness of display—for curation versions, we split composite attributes to facilitate detailed editing, while release versions present them in their grouped form.

**Merging schema**. A merging schema table summarizes how original fields/attributes were harmonized into curated fields/attributes and records their completeness and variability before (‘original_’) *and after* (‘*curated_*’) harmonization. For cMD, a single merging schema table contains six columns: original_field, original_field_completeness, original_field_unique_values, curated_field, curated_field_completeness, and curated_field_unique_values. For *cBioPortalData*, the schema is structured similarly but accounts for the many-to-many mapping relationships between original and curated attributes.

***OmicsMLRepoR* package implementation**. The *OmicsMLRepoR* Bioconductor package enables users to navigate harmonized metadata through ontological relationships in a robust and user-friendly manner. The main function, tree_filter, performs searches that include not only the queried terms but also their synonyms and all descendant terms, leveraging the ontology hierarchies we established during harmonization.

To address use cases where users need subsets of multi-valued and composite attributes, *OmicsMLRepoR* provides metadata reshaping functions—spreadMetaTb and gatherMetaTb. These functions spread or gather a metadata table anchored around selected attributes, transforming between wide and long formats to enhance downstream analysis. This design allows users to maintain both sample-focused and attribute-focused analytical workflows from the same underlying harmonized data structure.

## Results

We harmonized experimental metadata from 212 027 samples across 468 studies available through the cMD and cBioPortalData packages (Fig. 1A). The quality of harmonized metadata was enhanced through de-replication, ontology incorporation, and reconciliation (Supplementary Table 6). We assessed the improved metadata quality and the impact of harmonization using four criteria (Fig. 1B): 1) attribute standardization, representing the consolidation of variables and measured as the number of original attributes merged into each new curated attribute (compression); 2-1) value standardization, measured as the number of unique values per attribute (consolidation); 2-2) value correction, represented as the proportion of values updated during curation (correction rate); and 3) the combined impact of attribute and value standardizations, demonstrated through the completeness of the attributes (completeness), represented as the proportion of the non-*NA* values per attribute.

### Augmented metadata quality for *cMD*

**Figure 2:**
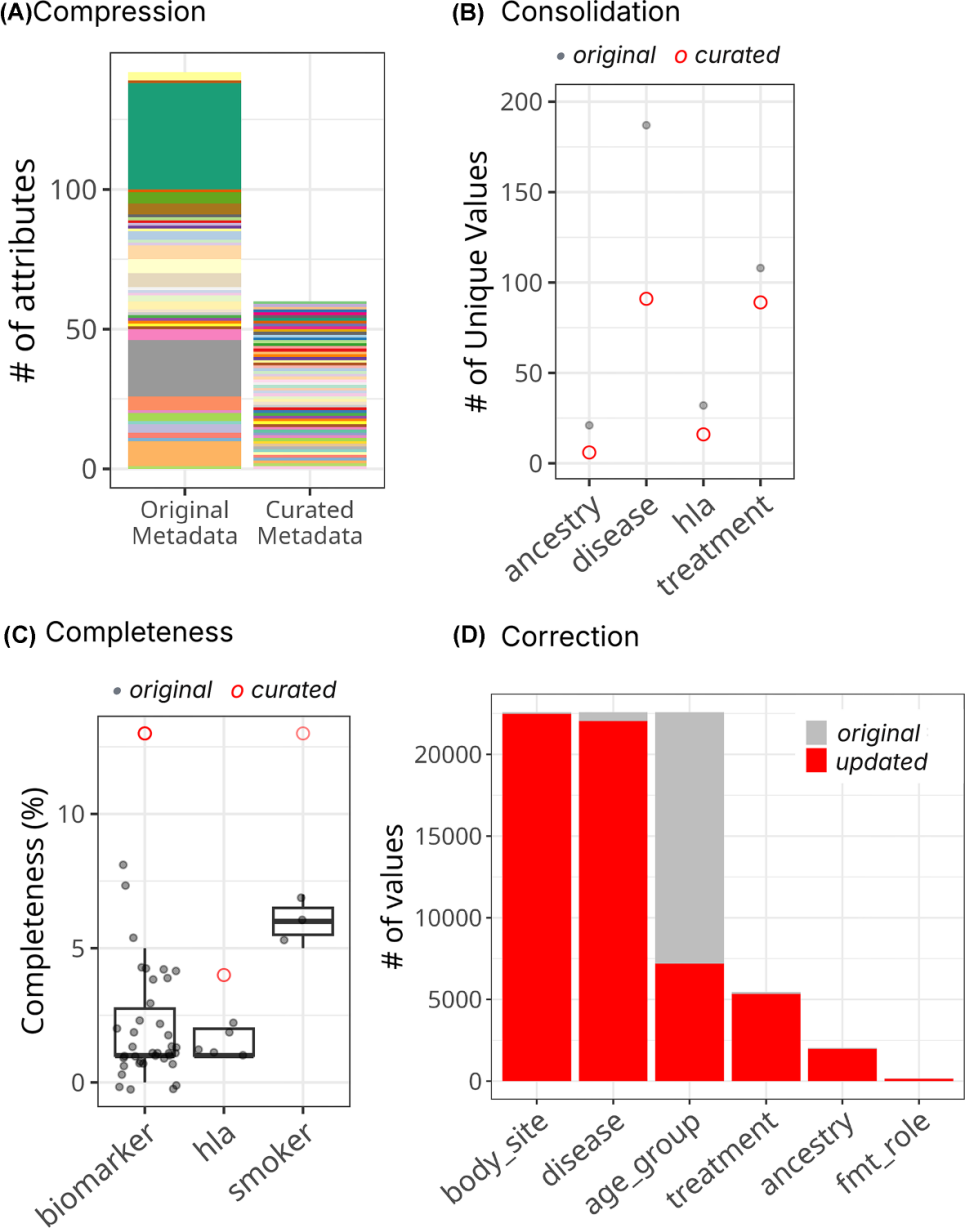
Improved metadata quality for *curatedMetagenomicData*. (A) Attribute compression. The left bar (‘Original Metadata’) displays 142 original attributes colour-coded by the harmonized attribute(s) into which they were consolidated. The right bar (‘Curated Metadata’) shows the resulting 66 harmonized attributes. Each colour represents the mapping relationship between original and curated fields. The dramatic height reduction illustrates the consolidation of redundant and dispersed information. (B) Value consolidation across key attributes. For each curated attribute (x-axis), grey points indicate the number of unique values in original attributes, while red circles show the number of unique values in the harmonized attribute. The dramatic reduction in unique values demonstrates successful standardization through ontology mapping. (C) Improved completeness through strategic consolidation. Grey points represent completeness of each original attribute, while red circles indicate completeness of harmonized attributes (x-axis). The curated attributes show substantially higher completeness by unifying information previously dispersed across multiple sparse columns. (D) Correction rate quantifies curation intensity. For each attribute, the grey bar shows values preserved in their original form, while the red bar indicates values modified during harmonization. The high correction rates reflect the extensive use of non-standardized terminologies in the original metadata, while lower correction rates indicate these fields were already relatively well-standardized.

After harmonization, 142 original attributes were reduced to 66 (Fig. 2A). The biomarker achieved the greatest data compression, as 38 original attributes across different biomarkers were merged into a single composite attribute, ‘*biomarker*’. The harmonized metadata improves the completeness of individual attributes (Fig. 2B). For example, the curated ‘*hla*’ column reached ∼5% complete, while each of the five original columns about *hla* genotypes (*HLA, hla_dqa11, hla_dqa12, hla_drb11*, and *hla_drb12*) was <2% complete. We observed a very high correction rate in cMD harmonization because most of the categorical variables used non-standardized terms. For example, >97% of values in the original *disease* and *treatment* columns were mapped to ontology terms in their curated columns, thereby improving interpretability, findability, and interoperability (Fig. 2C and Table 3). The cMD metadata usability was further improved by reducing redundancy, correcting mismatches, reorganizing values under proper attributes, and fixing typos. Our harmonization process also untangles intertwined and buried information. For example, the original cMD metadata had two poorly defined attributes (*study_condition* and *disease*) that contained mixed information. The harmonized schema separates these into three clearly defined attributes (*control, target_condition*, and *disease*), enabling users to identify unconventional situations such as non-healthy participants serving as controls or differentiate two participants with the same conditions (e.g. having both type 2 diabetes and adenoma) subjected to different studies (e.g. studies targeting type 2 diabetes versus adenoma), which will require distinct interpretations.

**Table 3:** Examples of original and curated values.^a^

Original *treatment* column	Curated *treatment* column
antilipid;antihta;antidiab;diuranse;ace_inhib; beta_blockers;ca2_cbl;metformin;glp_1; dppiv;insulin;statin;asa;clopidogrel; nitrate;ppi;xantoxinh	Antilipidemic Agent;Antihypertensive Agents;Anti-diabetic Agent;Diuretic;Angiotensin-Converting Enzyme Inhibitors;Beta-Adrenergic Antagonist;Calcium Channel Blocker;Metformin;glucagon-like peptide-1 receptor agonist;Dipeptidyl-Peptidase IV Inhibitors;Insulin;statin;Aspirin;Clopidogrel;Nitrate;Proton Pump Inhibitor;Xanthine Oxidase Inhibitor
metformin;sitagliptin;lantus;solostar;novorapid	Metformin;Sitagliptin;Insulin Glargine;Insulin Aspart

a The curated treatment column (on the right) contains more detailed and accurate information than the original treatment values (on the left). The standardization involves re-recording values (e.g. brand names such as ‘Lantus Solostar’, which were initially entered separately as ‘lantus’ and ‘solostar’) into a raw generic drug (e.g. ‘Insulin Glargine’). These are examples from the cMD.

### Augmented metadata for cBioPortal

**Figure 3:**
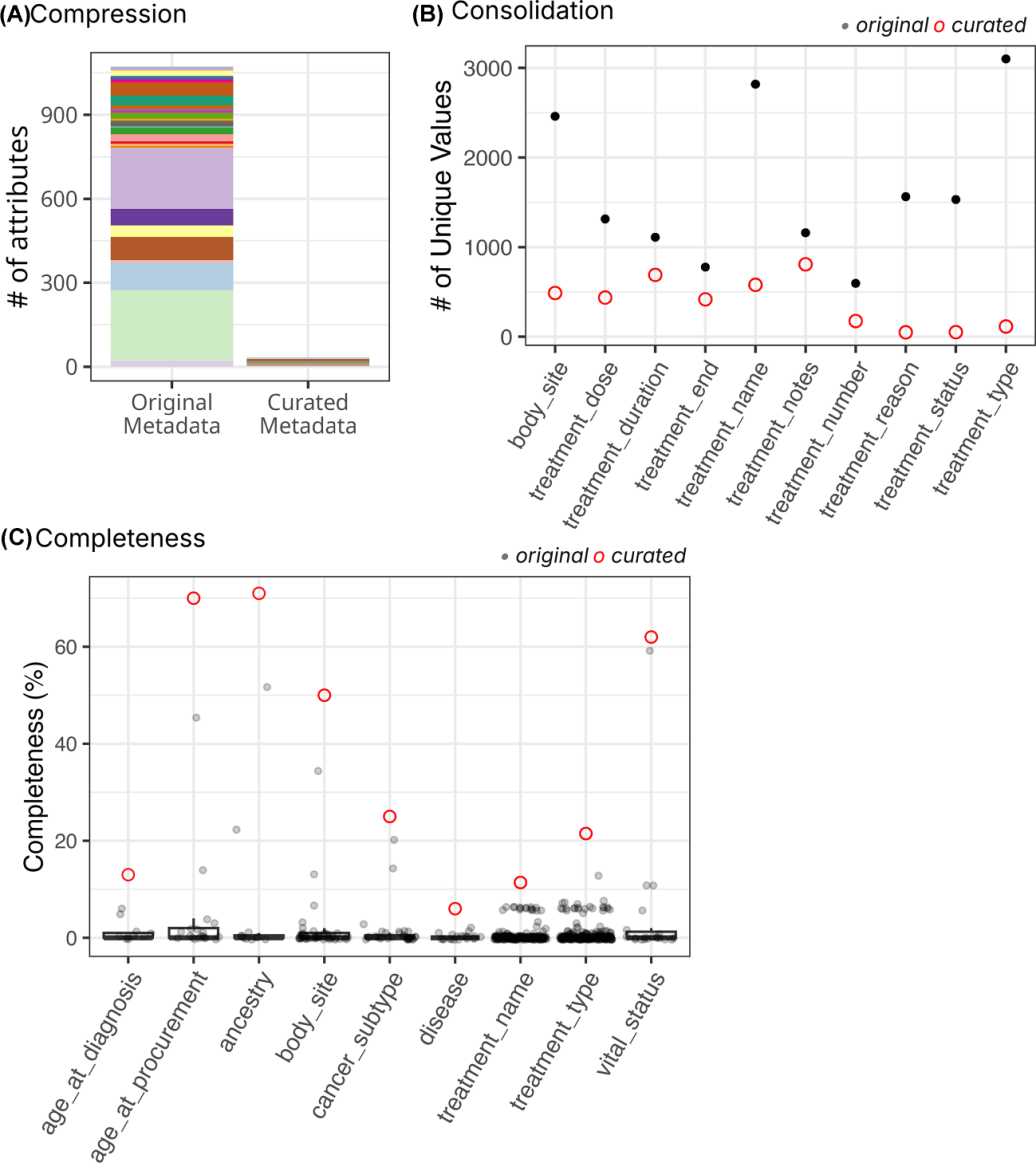
Improved metadata quality for *cBioPortalData*. (A) The left bar (‘Original Metadata’) represents 673 original attributes selected for harmonization based on completeness, clinical relevance, and prevalence across studies. Colours indicate which harmonized attribute(s) each original field contributed to, revealing the many-to-many mapping relationships in cBioPortal harmonization. Multiple original attributes often contributed to the same curated field, while some original columns contained mixed information that was properly distributed across multiple curated attributes. The right bar shows 30 harmonized attributes. The total number of source attributes exceeds 673 because some original columns contributed to multiple curated attributes during the separation of inappropriately merged information. (B) Top 10 attributes with greatest value consolidation. Grey points represent counts from individual original attributes, while red circles indicate the harmonized attribute. (C) Top 10 attributes with greatest completeness improvement. Grey points show completeness of original attributes; red circles indicate harmonized attributes (x-axis).

The snapshot of cBioPortal sample metadata used for harmonization contained 189 439 samples, ranging from 5 to 25 775 samples per study, from 375 studies, including TCGA, TARGET, and publications from individual labs. These samples’ metadata contained 3733 attributes, with >95% attributes having <4% data completeness. We harmonized 673 columns containing information on core attributes (i.e. age, body site, sex, disease, treatment, and ancestry) into 30 curated attributes (Fig. 3A). The maximum data compression was achieved for treatment types, where values across 254 original attributes were condensed into a single curated attribute, *treatment_type* (Supplementary Table 2). During the initial round of harmonization, we observed an average of ∼71% reduction in the number of unique values for curated attributes (Fig. 3B). The curated attributes exhibit significantly improved completeness (Fig. 3C). About 78% of the original attributes (527 out of 673) were <1% complete; however, the harmonized attributes have an average completeness of ∼25%, and half of the attributes (15 out of 30) have a completeness >10%. cBioPortal’s sample metadata harmonization involved a many-to-many merging process, where one original column frequently contributed to multiple curated columns and vice versa; therefore, our ‘correction rate’ measure was not applicable.

### Analysis of harmonized metadata

**Figure 4:**
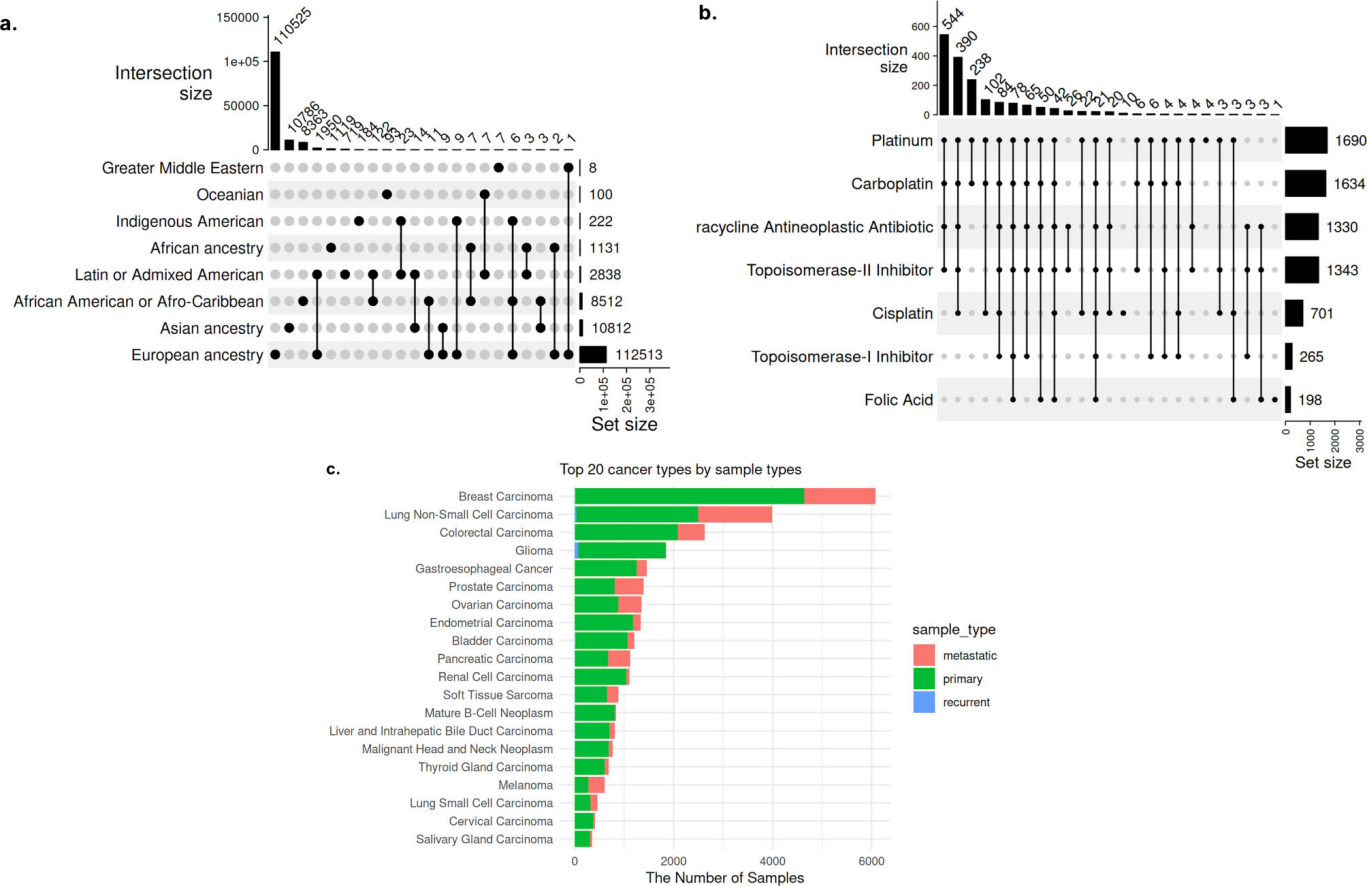
Repository-wide statistics enabled by harmonized metadata. (a) Comprehensive ancestry distribution across cBioPortal studies. UpSet plot showing the distribution of 134 032 samples from 218 studies across eight standardized ancestry categories and their combinations. The top bar chart displays intersection sizes. The matrix below shows which ancestry categories are present in each intersection (filled circles), with connecting lines indicating combinations.(b) Treatment landscape for ovarian carcinoma across studies. This plot summarizing treatment names for 1817 ovarian carcinoma samples from four independent studies, enabled by the harmonized treatment_name attribute that consolidated information from 254 original treatment-related columns. The numerical labels indicate sample counts for each treatment, while the x-axis shows set sizes for treatment combinations. (c) Sample type distribution across top 20 cancer types. Stacked bar chart showing the availability of primary, metastatic, and recurrent samples across cancer types, based on harmonized sample_type attribute.

The following examples illustrate repository-wide analyses enabled by harmonized metadata:

***Ancestry***. Approximately 71% of cBioPortal samples (*n* = 134 032) contain information related to ethnicity, race, and ancestry, which were spread across 11 attributes (Supplementary Table 2). We harmonized these into two attributes—ancestry and ancestry_details. The ancestry attributes comprise eight child terms of the ‘ancestry category’ (HANCESTRO:0004) ontology term, yielding 25 unique combinations of ethnic backgrounds (Fig. 4a). Any values with additional details beyond these eight child terms were mapped to ontologies (HANCESTRO and NCIT) and saved under the ancestry_details column. This harmonized view enables researchers to quickly identify available data for population-specific analyses and highlights critical diversity gaps requiring future data collection efforts.

***Treatment names***. Using the harmonized cBioPortal metadata, we can access samples across studies from a single query. For example, we searched all treatment names to which ovarian cancer samples were exposed and found 1817 ovarian carcinoma samples from four studies that contained treatment name information. Harmonized data show that platinum is most common treatment, frequently combined with carboplatin, tetracycline neoplastic antibiotic, and topoisomerase II inhibitor, as well as other complex multi-drug regimens (Fig. 4b). This cross-study synthesis would be extremely difficult with the original heterogeneous metadata, where identical drugs were recorded using variant brand names, abbreviations, and generic names across different studies.

***Sample and specimen types***. In the original cBioPortal metadata, sample- and specimen-type information was spread across >50 attributes. We harmonized the top three most complete original attributes (SAMPLE_TYPE, SAMPLE_CLASS, and SPECIMEN_TYPE) that were redundant and also mixed with other features, such as disease name and treatment type. In the harmonized metadata, the specimen_type attribute includes only standard terms for categorizing biological materials by their physical form and collection method, such as ‘body fluid specimen’ and ‘biopsy specimen’. The sample_type attribute categorizes biological samples based on their neoplastic characteristics, such as ‘Primary Neoplasm’ and ‘Metastatic Neoplasm’ (Fig. 4c). The 77 unique values under three original attributes were harmonized into 20 standardized terms under two curated attributes. For these two harmonized attributes, completeness actually decreased because values belonging to different/irrelevant attributes had been mixed together in the original data, artificially inflating completeness through the inclusion of irrelevant information. About 61% of the samples have sample_type information, and the majority (52%, *n* = 59 831) are classified as ‘Primary Neoplasm’, followed by ‘Neoplasm’ (30%, *n* = 34 998) and ‘Metastatic Neoplasm’ (17%, *n* = 19 892). The standardization enables researchers to rapidly identify studies with specific sample types for comparative analyses of primary versus metastatic disease, plan meta-analyses requiring matched sample types, or identify gaps where additional data collection is needed (e.g. limited metastatic samples for most cancer types). The colour-coded visualization would be impossible with the original metadata where sample types were mixed with disease names and treatment information across dozens of inconsistently named attributes.

***Vital status***. Approximately 62% (*n* = 117 950) of the samples in cBioPortal have vital status information initially spread across 20 different attributes, while there was one attribute ('OS_STATUS') with high completeness (59%). This harmonization consolidated 67 original values into three curated values (Dead, Alive, and Unknown), significantly improving consistency, findability, and interoperability.

### *OmicsMLRepoR* for accessing harmonized metadata

Controlled vocabularies (i.e. ontologies) are highly recommended for standardizing metadata descriptions, and several software tools support this implementation [47–50]. However, querying data through ontological relationships remains limited because most existing data resources use ontologies primarily for annotation rather than enabling flexible, hierarchy-aware queries. Furthermore, the functionalities of existing tools are often scattered, complex, and not user-friendly. To facilitate efficient use of our harmonized metadata, we developed the *OmicsMLRepoR* [51] Bioconductor package. It provides intuitive functions for querying harmonized metadata through ontological relationships, reshaping data for downstream analysis, and identifying shared ancestor terms.

## Discussion

We address critical challenges in omics data accessibility and reuse through comprehensive, retrospective, and cross-study metadata harmonization. We have harmonized key attributes across 468 studies encompassing >210 000 samples from major cancer genomics and metagenomic databases. Through manual harmonization guided by empirically derived schemas and quality control procedures, we demonstrate substantial improvements across multiple metadata quality dimensions: consolidated attributes (from 815 to 96), reduced unique values per attribute (71% average reduction), high correction rates (>97% for categorical variables), and increased completeness of major attributes. Our flexible attribute types (i.e. composite, multi-valued, and *_details attributes) enable practical metadata integration while preserving the granular information required for diverse analytical approaches. Our harmonized data directly address several pressing concerns in omics research. Insufficient representation of minorities in genomic sequencing projects risks widening health disparities, while failure to include sex as a biological variable in AI/ML projects may compromise model validity. Additionally, most cancer omics AI/ML applications typically integrate three to four major omics data types (e.g. genomic, transcriptomic, proteomic, and epigenomic) [33, 34], while the incorporation of patient-level and treatment-related data remains limited [7, 52]. Our standardized, machine-readable reporting of sample metadata helps address these critical gaps.

A persistent challenge in metadata management is achieving a balance between comprehensiveness and practicality, both during curation and usage. Manual curation often leads to redundancy as curators create new attributes when existing ones do not exactly match or when data schemas become too complex to navigate efficiently. This redundancy scatters related data across multiple attributes, making it difficult for users to locate relevant information. Rich, standardized metadata provide understandability and are critical to the FAIR principles—indeed, the research community increasingly treats well-curated datasets, alongside the publications that describe them, as primary research outputs. Comprehensive metadata schemas are, therefore, valuable and should be encouraged. Where we see opportunity for a complementary approach is in supporting users who must navigate the substantial body of legacy data that pre-dates the widespread adoption of such standards, where information is often dispersed across heterogeneous attributes and recorded with non-standardized vocabularies. By harmonizing this legacy data against established ontologies, we aim to make it more findable and integrable while the community continues to advance prospective standards for new data collection. Our approach aims to improve metadata accessibility and interoperability without sacrificing granularity, complementing established standardization frameworks (e.g. OMOP CDM and ISA) that are optimized for the data collection stage, while supporting retrospective harmonization of already-collected research data. In particular, the flexibility of our approach can facilitate the integration of heterogeneous data and enable quick adaptation to evolving standards as new measurement techniques or data collection practices emerge.

Manual curation of legacy datasets will remain necessary for the foreseeable future, and we view our work as a contribution to this problem rather than as an alternative to scalable solutions. Recent automated and hybrid approaches—particularly those that incorporate community standards such as MIAME, MINSEQE, and MIxS into generative AI workflows [24, 25]—offer a scalable path forward, and we expect them to play an important role in metadata harmonization. At the same time, manual curation provides ground-truth data, critical for validating automated outputs, and the contextual judgement needed for non-standard formats and ambiguous definitions. Quantitative assessments illustrate why both automated and manual harmonization tracks are needed: three-person expert teams required 4 weeks to curate 22 000 samples [53], and our own harmonization required >1500 person-hours, underscoring that purely manual approaches cannot scale to repositories containing millions of records, while purely automated approaches still require expert validation and quality control at every stage. Our large-scale manually curated dataset, with its comprehensive provenance tracking and documented decision logic, is intended to provide immediately usable harmonized metadata as well as to support the development of automated solutions by providing training and validation data for automated systems, and quality benchmarks against which automated outputs can be evaluated. The OmicsMLRepo data schema could also serve as a prospective standard for future omics data collection, bridging retrospective harmonization and prospective standardization. We see manual and automated harmonization as complementary rather than competing, with manually curated resources like ours providing the high-quality validation resource that automated methods will continue to need.

## Supplementary Material

baag027_Supplemental_File

## Data Availability

The *OmicsMLRepoR* package is distributed through Bioconductor (https://www.bioconductor.org/packages/release/bioc/html/OmicsMLRepoR.html). The harmonized cMD and cBioPortal metadata tables are available in Zenodo (https://zenodo.org/record/14110745) and can be accessed directly through the *OmicsMLRepoR* package. The data merging schemas and data dictionaries are provided in the supplementary tables.
