# Supplementary material for: Large-scale manual curation and harmonization of metadata from metagenomic and cancer genomic repositories: challenges and solutions
Source: Database (Oxford). 2026 May 22;2026:baag027. doi: 10.1093/database/baag027 (PMC13196698; doi:10.1093/database/baag027)
Supplement: baag027_Supplemental_File [file baag027_supplemental_file.pdf]

Supplementary Tables

Supplementary Table 1. Merging schema for *cMD*. The merging schema table includes the name, completeness (`*_completeness` columns, a percentage of non-missing values), and variability (`*_unique_values` columns, a number of unique values) of both original and curated fields, as well as the number of original fields used for each curated field (`original_field_num`). When multiple fields are involved, they are separated by semicolons (;).
https://github.com/waldronlab/OmicsMLRepoData/blob/master/inst/extdata/cMD_merging_schema.csv

Supplementary Table 2. Merging schema for *cBioPortalData*
https://github.com/waldronlab/OmicsMLRepoData/blob/master/inst/extdata/cBioPortal_merging_schema.csv

Supplementary Table 3. Data dictionary for *cMD*
https://github.com/waldronlab/OmicsMLRepoData/blob/master/inst/extdata/cMD_data_dictionary.csv

Supplementary Table 4. Data dictionary for *cBioPortalData*
https://github.com/waldronlab/OmicsMLRepoData/blob/master/inst/extdata/cBioPortal_data_dictionary.csv

Supplementary Table 5. Definition of data dictionary columns

Column_Name	Description
ColName	The name of the field/column/attribute in the dataset.
ColClass	The data type of a given field/column/attribute. It can be an integer, character, or boolean.
Unique	The uniqueness constraint. TRUE if it should be unique.
Required	Required/optional status. TRUE if the field/column/attribute must have a value ("required").
MultipleValues	Whether multiple values can be assigned to a given attribute. TRUE if multiple values can be assigned.
Description	A detailed explanation of what the field/column/attribute represents and how it should be used.
AllowedValues	Allowed values for the given column/attribute. If 'dynamic_enum' contains a value, all the terms satisfying the 'dynamic_enum_property' of the 'dynamic_enum' value are allowed.
Delimiter	A symbol used to separate multiple attributes under a composite attribute
Separator	A symbol used to separate multiple values under a multi-valued attribute

DynamicEnum	An ontology term used for a dynamic enum. Any terms that have the specified property (under ‘dynamic_enum_property’) with this term are automatically accepted as ‘allowed values’.
DynamicEnumProperty	A property to propagate from the ontology term specified under the ‘dynamic_enum’ column. It can be children, descendants, parents, and ancestors. This is required if 'dynamic_enum' is assigned.

Supplementary Table 6. Summary of metadata quality issues identified across cMD and cBioPortal (cBP) repositories

Quality Issue Category	Repo	Prevalence / Scale	Example	Resolution Strategy
Non-standardized terminologies	cMD	>97% of disease and treatment values required ontology mapping	‘antihta' → 'Antihypertensive Agents'; 'lantus;solostar' → 'Insulin Glargine'	Manual ontology mapping
	cBP	~71% average reduction in unique values after standardization	Variant drug names, abbreviations, and brand names across studies for the same entity	Consolidation to ontology terms
Redundant/ dispersed attributes	cMD	142 original attributes compressed to 66 (54% reduction)	38 biomarker-related attributes merged into 1 composite attribute; 5 HLA columns merged into 1	Schema mapping with composite attributes
	cBP	673 attributes compressed to 30 (96% reduction); 3,733 total attributes with >95% having <4% completeness	254 treatment-related attributes (all or part of information) condensed into 1 curated `treatment_type` attribute	Many-to-many schema mapping
Low completeness due to dispersion	cMD	Individual HLA columns <2% complete; merged column ~5% complete	Information about the same concept is scattered across multiple sparse columns	Attribute consolidation
	cBP	78% of original attributes (527/673) were <1% complete; harmonized attributes average ~25% complete	Ancestry information is spread across 11 attributes; vital status across 20 attributes	Cross-attribute consolidation
Conflicting annotations	cMD	Cross-attribute logical inconsistencies identified	Disease-sex impossible combinations (e.g., 'prostate cancer' with 'female')	Cross-attribute logical consistency checks
	cBP	337 sex discordances identified; 303 from a single study (*luad_msk_npjpo_2021*)	Same patient sample with different sex annotations across studies	Systematic vs. random error distinction; majority rule for time-invariant attributes
Intertwined/ buried information	cMD	2 poorly defined attributes contained mixed information	`study_condition` and disease mixed control status, target condition, and disease information	Separation into 3 clearly defined attributes (`control`, `target_condition`, `disease`)
	cBP	Sample type attributes mixed with disease names and treatment types	`SAMPLE_TYPE`, `SAMPLE_CLASS`, `SPECIMEN_TYPE` contained 77 unique values, mixing neoplastic characteristics, specimen forms, and unrelated metadata	Separation into specimen_type and sample_type with 20 standardized terms
Typos and data entry errors	cMD	Identified through round-trip ontology validation	Misspelled drug names, incorrect abbreviation interpretations	Ontology ID/synonym lookup and comparison

	cBP	Identified through dynamic enum validation	Values not belonging to the allowed descendant terms of dynamic enumeration nodes	Validation against ontology-derived allowed value pools
Artificially inflated completeness	cBP	Completeness decreased for 2 harmonized attributes after removing irrelevant values	`specimen_type` and `sample_type`: original attributes appeared more complete because they included values belonging to other attributes	Strict attribute scoping during harmonization
